# Scrape cytology in the early diagnosis of eyelid sebaceous carcinoma

**DOI:** 10.4103/0970-9371.73301

**Published:** 2010-10

**Authors:** CA Arathi, C Vijaya

**Affiliations:** Department of Pathology, Sree Siddhartha Medical College, Tumkur, India

**Keywords:** Sebaceous carcinoma, eyelid, scrape cytology

## Abstract

The low incidence and the non-specific clinical symptoms led us to conclude that the diagnosis of a sebaceous carcinoma of the eyelid often occurs very late. Sebaceous carcinoma of the ocular adnexa is a malignant neoplasm which can exhibit aggressive local behavior, can have pagetoid spread and can metastasize to regional lymph nodes and distant organs. The neoplasm is known to masquerade as other benign and less malignant lesions, and has relatively high morbidity and mortality. Scrape cytology was done in a 70-year-old female with a tumor in left upper lid. Cytological smears were suggestive of sebaceous carcinoma. Subsequently, histopathology confirmed the diagnosis of sebaceous gland carcinoma. The article highlights the role of scrape cytology in early diagnosis and subsequent appropriate surgical management of eyelid sebaceous gland carcinoma, to prevent recurrence and metastasis.

## Introduction

Sebaceous carcinoma is a very rare malignant tumor primarily found in the area of the eyelid. Most of these carcinomas originate in the tarsal meibomian glands, although they may rarely originate in the glands of Zeis.[1] They are most lethal ocular adnexal tumors, second only to melanoma, frequently masquerading as other less aggressive eye lid lesions like chalazion, chronic blepharoconjunctivitis, kerato-conjunctivitis, basal cell carcinoma, and squamous cell carcinoma. Early diagnosis is thus of extreme importance to avoid high morbidity and mortality (23%).[2] Scrape cytology is a rapid, non-invasive, easy to perform and an inexpensive technique, the application of which is very helpful in the early diagnosis of surface ocular lesions. We are presenting a case of sebaceous carcinoma of the eyelid, diagnosed by scrape cytology, later confirmed by histopathology.

## Case Report

A 70-year-old female patient presented with an ulcerated tumor, measuring 3 cm in diameter, at the left upper lid, with a bloody encrusted lid margin and showed putrid secretion on the surface. The ocular examination revealed no conjunctival or corneal involvement. There were no lymph nodes. With the informed consent from the patient, after applying local anesthetic drops, with the help of spatula, the lesion was scraped gently, smeared and immediately immersed in 95% ethyl alcohol and stained with hematoxylin and eosin.

**Figure 1:**
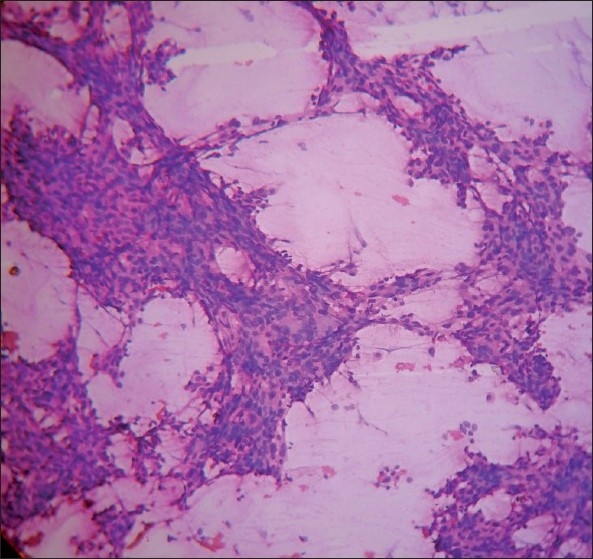
Highly cellular smear showing cohesive clusters of cells in a clear background (H and E, ×100)

**Figure 2:**
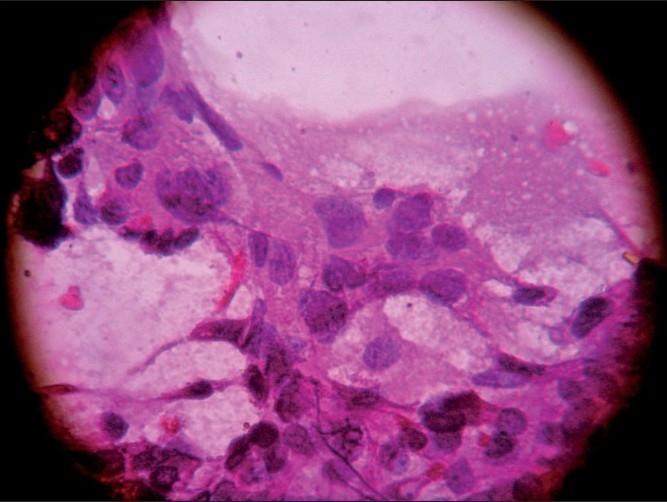
Cytomorphology of pleomorphic cells with round to oval large hyperchromatic nuclei. Focal glandular pattern is also seen (H and E, ×400)

**Figure 3:**
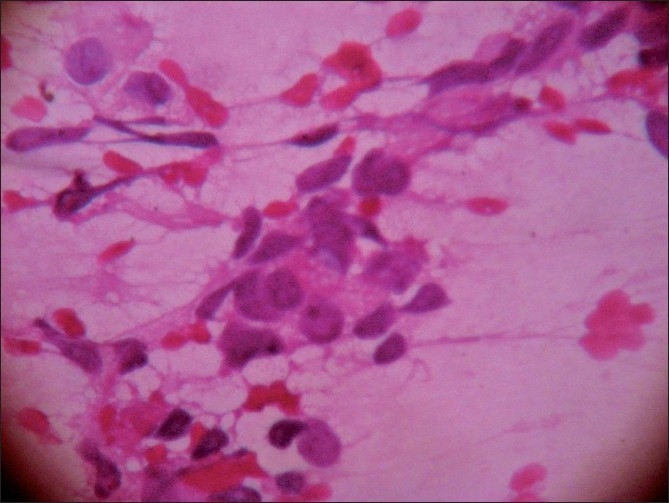
Smear showing micro vacuoles both in cytoplasm and in the background (H and E, ×400)

**Figure 4:**
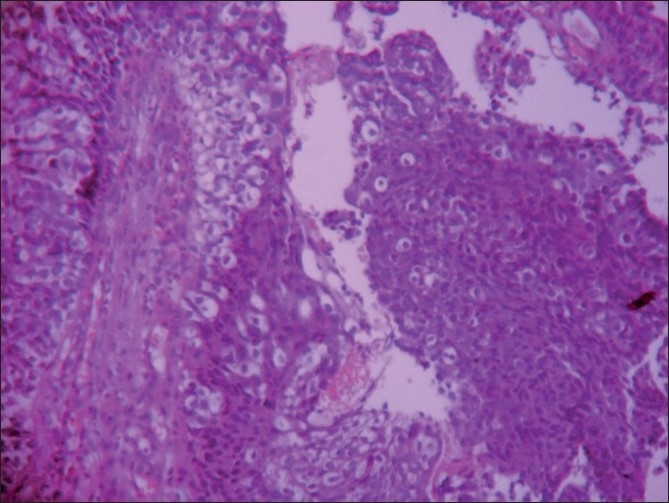
Section showing tumor cells arranged in lobules (H and E, ×100)

**Figure 5:**
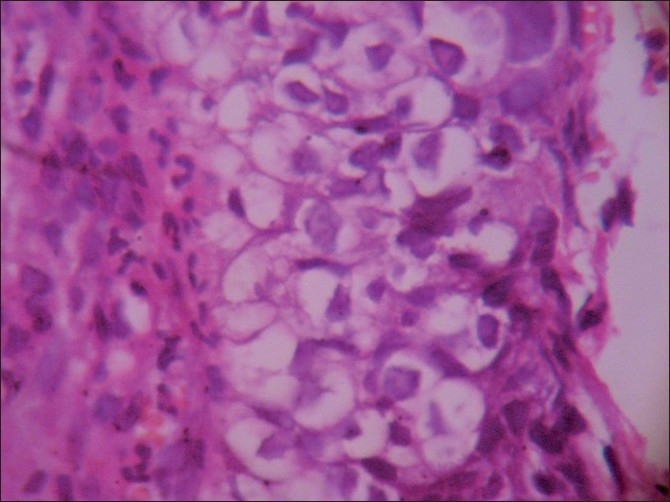
Malignant cells with sebaceous differentiation showing foamy cytoplasm (H and E, ×400)

The smears were highly cellular, arranged in sheets, three-dimensional clusters, acinar pattern and scattered singly. The cells were polygonal, having pleomorphic, hyperchromatic nuclei, and cytoplasm studded with micro vacuoles representing lipids [Figures 1–3]. Background was clear with a few lipid vacuoles. Based on these features, a cytological diagnosis of sebaceous carcinoma was made. Sections from the tumor tissue showed cells arranged in lobules. The cells were pleomorphic with basophilic nuclei, and had foamy cytoplasm; the cytoplasmic vacuoles were typically small [Figure 4 and 5].

## Discussion

Ocular surface lesions are easily accessible to the application of scrape cytology, which is a rapid, non-invasive, easy to perform and an inexpensive technique.[3] The method is reliable and does not require special instruments or set up.

Scrape cytology offers a better cell yield even in keratinising lesions and small focal lesions. It also offers good morphological details, if smear is fixed immediately. Results of scrape cytology are likely to be more specific and sensitive.[3] Regular application of scrape cytology to ocular surface lesions would elevate the confidence level and experience among the pathologists.[3]

Historically, sebaceous gland carcinoma of the eyelid is notorious for masquerading as a more common benign condition, often resulting in a long delay before the correct diagnosis is made. Such a delay in diagnosis can increase the chances of local recurrence and metastasis.[4] Clinically, it is often mistaken for a chalazion, and also, it may mimic many other conditions, hence called great masquerade. Recurrent chalazion, chronic, recalcitrant, atypical blepharitis or atypical unilateral papillary conjunctivitis should be considered for biopsy.[5]

Many ophthalmologists therefore submit recurrent chalazion to the pathologist with a clinical diagnosis of “rule out sebaceous carcinoma”, and suspect the infiltrative form of sebaceous carcinoma in patients with unilateral thickening of the eyelid with loss of eyelashes (blepharitis is typically bilateral condition).[6]

The incidence of sebaceous carcinoma in the western literature is reported to be less than 1% of all eyelid tumors and accounts for 1–5% of all malignant eyelid tumors. Recent studies from India and China have shown that sebaceous carcinoma accounts for 33–60% of malignant eyelid tumors. It thus seems that the incidence of sebaceous gland carcinoma has a geographical variation and is more common in Asian population.[4]

Cytological features, especially cytoplasmic vacuolation which is the diagnostic feature in sebaceous carcinoma, are very helpful in the early diagnosis. It can be associated with extensive pagetoid spread within the conjunctival epithelium. If the sebaceous carcinoma is unknown, then pagetoid spread can easily masquerade as *in-situ* squamous cell carcinoma of conjunctiva, unless the pathologist detects the vacuolated tumor cells.[3]

In a few cases of sebaceous carcinoma, the fatty contents liberated from sebaceous glands due to ductal obstruction might evoke a granulomatous response accompanied by neutrophils, creating confusion with blepharitis and chalazion.[7] However, features of extracellular accumulation of fat, plasma cells, lymphocytes, occasional multinucleated giant cells suggest the diagnosis of chalazion, and the presence of non-granulomatous inflammation, often containing neutrophils, suggest the possibility of blepharoconjunctivitis.

The other differential diagnosis includes pilomatrixoma which shows bland sheets of basaloid cells, nucleated basophilic cells and “ghost” cells. Basal cell carcinoma shows less cellular smears and tightly cohesive small clusters of monomorphic basaloid cells without vacuolation.[2]

Sebaceous carcinomas of eyelid are characterised by a high degree of biological aggression concerning local recurrence and metastasis; hence, early diagnosis is very essential.

We are presenting this case to highlight the role of scrape cytology in early diagnosis and subsequent appropriate surgical management of eyelid sebaceous gland carcinoma, to prevent recurrence and metastasis.
